# From Prediction to Mechanism: Explainable AI for Plant–Microbiome Multi-Omics Integration and Biological Discovery

**DOI:** 10.3390/ijms27198615

**Published:** 2026-09-26

**Authors:** Lupin Deng, Lei Liu, Yu Luo, Mengdi Dai, Ziran Ye, Xingguo Xiong, Yuxiang Li, Dedong Kong, Xiangfeng Tan

**Affiliations:** 1 Institute of Digital Agriculture, Zhejiang Academy of Agricultural Sciences, Hangzhou 310021, China; 14777145769@stu.gxnu.edu.cn (L.D.);; 2 Key Laboratory of Ecology of Rare and Endangered Species and Environmental Protection (Ministry of Education), Guangxi Normal University, Guilin 541006, China; 3 Institute of Rural Development, Zhejiang Academy of Agricultural Sciences, Hangzhou 310021, China; 4 Agricultural Artificial Intelligence Innovation Center, Zhejiang Academy of Agricultural Sciences, Hangzhou 310021, China

**Keywords:** explainable artificial intelligence (XAI), plant-microbiome interactions, multi-omics integration, mechanistic discovery, causal inference, plant holobiont

## Abstract

Predicting complex phenotypes from plant-microbiome multi-omics data has advanced significantly with machine learning and deep learning. However, traditional black-box models remain limited by their focus on predictive performance rather than mechanistic biological understanding. To bridge this gap, explainable artificial intelligence (XAI) has emerged as a key framework to transform biological data analysis from correlation mapping to hypothesis-driven discovery. This review synthesizes current advances in XAI methodologies, including post hoc feature attribution techniques and intrinsically interpretable neural architectures, and their applications in plant-microbiome interactions. We highlight how XAI enables multi-scale insights by mapping model predictions to functional genes, metabolic pathways, and microbial interaction networks. Furthermore, we address critical challenges in distinguishing statistical importance from true biological causality and advocate for a closed-loop framework that continuously integrates computational reasoning with wet-lab experimental validation. Finally, we explore future avenues, including biologically informed neural networks, causal AI, and multi-omics knowledge graphs. By facilitating the transition from predictive analytics to biologically interpretable and experimentally testable hypotheses, XAI may contribute to mechanistic discovery in plant-microbiome systems.

## 1. Introduction

### 1.1. The Plant Holobiont as a Biological Paradigm

Plant performance is increasingly recognized as an emergent property of dynamic interactions among the host, its associated microbiota, and the surrounding environment rather than a consequence of the plant genome alone. This perspective has established the plant holobiont as a central biological framework for understanding plant growth, nutrient acquisition, stress adaptation, and disease resistance, emphasizing that these traits arise from coordinated host-microbiome interactions across multiple organizational levels [1,2]. Consequently, deciphering plant phenotypes requires an integrated view of the molecular and ecological processes that collectively shape holobiont function.

Advances in high-throughput sequencing and multi-omics technologies have transformed our ability to investigate these complex interactions. The integration of host genomics, microbiome profiling, transcriptomics, metabolomics, phenomics, and environmental information has shifted plant-microbiome research from descriptive community characterization toward systems-level analyses of host–microbe interactions [3,4,5]. More recently, holo-omics has further expanded this framework by enabling the simultaneous analysis of biological processes across multiple molecular layers, providing unprecedented opportunities to explore the regulatory networks underlying plant phenotypes [2,6].

Despite these technological advances, the principal challenge is no longer generating biological data but extracting mechanistic knowledge from increasingly complex datasets [7,8]. Modern multi-omics studies routinely capture extensive information describing hosts, microbial communities, and their environments, yet translating these heterogeneous observations into experimentally testable biological hypotheses remains difficult. As the scale and complexity of plant-microbiome datasets continue to increase, the fundamental question has shifted from how to generate multi-omics data to how to infer biologically meaningful and mechanistically informative knowledge from them.

### 1.2. Why Biological Inference Remains the Major Bottleneck

The rapid expansion of multi-omics datasets has fundamentally shifted the bottleneck in plant-microbiome research from data acquisition to biological inference [9]. Although diverse omics technologies now provide comprehensive descriptions of host genetics, microbial communities, environmental conditions, and plant phenotypes, transforming these heterogeneous datasets into mechanistic understanding remains a major challenge. This difficulty arises from four interconnected barriers spanning data representation, biological interpretation, experimental validation, and ecological generalization.

The primary challenge lies in the intrinsic complexity of plant-microbiome multi-omics data. These datasets are highly heterogeneous, high-dimensional, compositional, and environmentally dependent, making robust data integration and signal extraction substantially more difficult than in conventional single-omics analyses [6]. Consequently, statistical associations may reflect technical variation or ecological context rather than biologically meaningful relationships.

A more fundamental challenge concerns biological interpretation. Most computational models are designed to identify predictive patterns, yet prediction alone rarely explains how host-microbiome interactions regulate plant phenotypes [10]. Because plant performance emerges from dynamic cross-kingdom interactions involving hosts, microorganisms, and environmental factors, statistically important features do not necessarily represent biologically causal processes [11].

Even when biologically plausible hypotheses are generated, experimental validation remains indispensable for establishing mechanisms. Computational predictions must ultimately be verified through perturbation-based experiments to distinguish causal regulators from correlated observations [12,13]. Furthermore, biological knowledge derived from controlled datasets often fails to generalize across diverse soils, climates, host genotypes, and agricultural management practices, limiting its practical application in complex field environments [14,15].

Together, these challenges highlight that the next generation of computational approaches must move beyond predictive accuracy toward models capable of producing biologically interpretable, experimentally testable, and environmentally robust knowledge.

### 1.3. From Explainability to Mechanistic Biology

Addressing these challenges requires a fundamental shift in how artificial intelligence is applied to plant-microbiome research. While machine learning (ML) has substantially improved the integration of complex multi-omics datasets and the prediction of plant traits, predictive performance alone provides limited biological insight [16]. A model may accurately identify microbial signatures associated with disease resistance or nutrient use efficiency yet fail to explain the biological processes underlying these predictions. Thus, the central objective of artificial intelligence (AI) is no longer simply to improve prediction, but to generate biologically meaningful and experimentally testable hypotheses.

This distinction highlights an important conceptual hierarchy. Prediction identifies statistical relationships between biological variables, whereas explainability seeks to reveal how computational models derive these relationships. Biological interpretation further evaluates whether model explanations correspond to known molecular, physiological, or ecological processes. Mechanistic understanding, however, requires evidence that links biological perturbation to downstream regulatory responses and ultimately to phenotypic outcomes. Explainable artificial intelligence (XAI) can help bridge this gap by prioritizing candidate genes, microbial taxa, metabolites, and interaction networks for biological interpretation and subsequent experimental testing [11,17]. Nevertheless, explainability should not be equated with mechanism, as model explanations remain dependent on data quality, model assumptions, and computational architecture.

To further narrow the gap between correlation and mechanism, increasing attention has been directed toward causal AI, which seeks to infer directional biological relationships rather than statistical associations alone. Although its application to plant-microbiome systems is still emerging, integrating explainability with causal reasoning provides a promising framework for identifying experimentally actionable hypotheses and improving the biological reliability of AI-assisted discovery [18]. Rather than replacing experimental biology, these approaches are expected to strengthen the iterative cycle between computational inference and experimental validation.

In this Review, we examine how explainable and causal AI can facilitate the transition from predictive modelling to mechanistic discovery in plant-microbiome multi-omics. We first discuss the biological and computational challenges underlying multi-omics integration, then critically evaluate recent advances in explainable AI and their applications in plant-microbiome research, and finally highlight future opportunities for developing biologically interpretable, causally informed, and experimentally actionable AI frameworks for sustainable crop improvement. Ultimately, we argue that the defining role of explainable AI is not to explain algorithms, but to make biological discovery increasingly transparent, testable, and mechanistically grounded. To distinguish established evidence from methodological opportunities, we differentiate throughout this review between approaches that have been directly applied to plant-microbiome systems, approaches demonstrated in plant biology more broadly, and architectures primarily established in other biological domains. The latter are discussed as transferable methodological frameworks rather than as established solutions for plant-microbiome research.

Several recent reviews have discussed plant-microbiome interactions [9,19], holo-omics [2], machine learning for multi-omics integration [20,21,22]. These studies provide important foundations for understanding the biological and computational landscape. However, less attention has been devoted to systematically connecting XAI-derived feature attribution with biological contextualization, experimental validation, causal inference, and iterative model refinement specifically in plant-microbiome systems. This review therefore focuses on the evidence chain linking prediction, explanation, biological hypothesis generation, and experimental validation, while distinguishing established plant-microbiome applications from methodological advances originating in other biological domains.

Relevant literature was identified through searches of major scientific databases, including Web of Science, PubMed, Scopus, and Google Scholar, using combinations of keywords related to plant-microbiome interactions, multi-omics, artificial intelligence, machine learning, deep learning, and explainable AI. Studies were selected based on their relevance to AI-assisted analysis, integration, interpretation, or biological discovery in plant-microbiome systems. Representative studies from plant biology and other biological domains were also considered when they provided methodological insights applicable to plant-microbiome multi-omics. Studies were excluded when they were unrelated to the review scope or lacked sufficient methodological or biological information for meaningful evaluation.

## 2. AI for Plant-Microbiome Multi-Omics Integration

### 2.1. Machine Learning for Phenotype Prediction and Biomarker Discovery

ML has emerged as a powerful approach for integrating heterogeneous plant-microbiome multi-omics data and linking molecular and microbial features to plant phenotypes across diverse biological contexts. One particularly active application is the prediction of plant disease resistance, for which classical algorithms such as Random Forest (RF), Support Vector Machine (SVM), and eXtreme Gradient Boosting (XGBoost) have been widely applied. By accommodating high-dimensional data and nonlinear relationships, these methods can improve phenotype prediction and facilitate the prioritization of candidate features associated with disease resistance.

Recent studies further demonstrate that incorporating biological prior information, such as kinship relationships, can substantially improve predictive performance. For example, ML models integrating kinship matrices achieved high prediction accuracy across multiple crop diseases, reaching up to 95% for rice blast and showing strong performance for rice sheath blight, rice black-streaked dwarf disease, wheat blast, and wheat stripe rust [23].

ML has also been applied to genome-wide functional annotation of plant resistance genes. The R-Predictor pipeline integrates multiple functional annotation modules, which uses protein language-model embeddings and systematically evaluates several downstream ML algorithms for LRR-domain prediction [24]. RF achieved the highest performance among the tested models. When combined with transcriptomic profiles from grapevine infected with gray mold and downy mildew, R-Predictor identified dozens of upregulated candidate resistance genes and outperformed existing tools in the identification of pattern recognition receptors. These findings demonstrate that ML-assisted annotation pipelines can expand the repertoire of candidate resistance genes and support their subsequent functional characterization [24].

For microbiome classification and host phenotype inference, RF and SVM have also proven effective for distinguishing microbial community states associated with plant health and environmental stress. A multi-omics study of 198 soybean accessions exposed to drought employed RF and XGBoost, together with SHAP (Shapley Additive explanations) model interpretation, and outperformed linear mixed models in capturing nonlinear relationships among host, microbial, metabolic, and environmental variables [25]. SHAP analysis identified environment-dependent biomarkers, including the isoflavone derivative daidzin and the archaeon *Candidatus Nitrosocosmicus* under drought conditions. SHAP interaction analysis further revealed cross-omics associations among daidzin, gamma-aminobutyric acid (GABA), and *Paenibacillus*, with GABA exhibiting high network connectivity. These results illustrate how ML-based multi-omics integration, when coupled with interpretable approaches, can identify stress-associated cross-kingdom interaction modules and prioritize candidate targets for crop improvement.

For biomarker discovery, embedded regularization methods such as least absolute shrinkage and selection operator (LASSO) and Elastic Net enable simultaneous feature selection and model regularization, making them particularly useful for high-dimensional datasets. Complementing these approaches, DNNGP, a deep neural network-based genomic prediction framework, integrates multi-omics information across crop species and outperforms GBLUP and several conventional prediction methods [26]. In addition, the SVM-based predictor ASRpro uses k-mer-derived features to identify abiotic stress-responsive proteins, achieving approximately 75–86% prediction accuracy [27].

Collectively, these studies demonstrate the capacity of ML approaches to connect genomic, transcriptomic, metabolic, and microbial features with host phenotypes across disease and abiotic-stress contexts. Nevertheless, many conventional ML frameworks depend heavily on predefined feature representations and may have limited capacity to learn hierarchical and highly complex nonlinear relationships directly from heterogeneous multi-omics data. These limitations have motivated the increasing use of deep learning approaches, which can automatically learn informative representations and model complex cross-omics dependencies.

### 2.2. Deep Learning and Multimodal Integration

The application of deep-learning architectures to plant-microbiome multi-omics should be considered in the context of a still-developing evidence base. Some architectures have already been used in plant or plant-associated microbiome studies, whereas others have been developed and validated primarily in human genomics, cancer biology, or general computational biology. We therefore distinguish demonstrated plant-microbiome applications from architectures presented primarily as transferable methodological frameworks when discussing these approaches. Although traditional machine-learning frameworks are effective for biomarker selection and cohort classification, their performance can be constrained when modeling the heterogeneous, high-dimensional, and strongly nonlinear relationships characteristic of plant-microbiome systems [28]. Deep learning (DL) addresses some of these limitations by learning hierarchical feature representations directly from complex data and has therefore become an increasingly important framework for multi-omics integration [25,29]. Within this framework, the stage at which information from different omics layers is combined—commonly categorized as early, intermediate, or late fusion—substantially influences the biological information retained by the model, the interactions it can learn, and its predictive performance [30].

Early fusion is the most straightforward strategy, in which feature matrices from different omics layers, such as plant gene expression, microbial abundance, and metabolite profiles, are concatenated into a single high-dimensional input and subsequently processed by a unified neural network [20,31]. This approach is simple to implement and allows the model to learn cross-modal relationships from the input stage. However, direct concatenation can amplify the challenges associated with high dimensionality, heterogeneous feature distributions, and modality imbalance. For example, a high-dimensional omics layer such as transcriptomics may dominate model training relative to a lower-dimensional modality such as metabolomics, potentially obscuring biologically informative signals from the latter [5,32]. Early fusion may also be sensitive to noise and missing data because modality-specific structure is not explicitly modeled before integration. Consistent with these limitations, early-fusion models based on AutoEncoder and Neural Network, have underperformed more structured integration strategies in several multi-omics classification benchmarks [33].

Intermediate fusion was developed to address these limitations by separating modality-specific representation learning from cross-modal integration [20,34]. In this framework, each omics layer is first processed by a dedicated encoder or subnetwork to generate a compact latent representation, after which the modality-specific representations are integrated within one or more shared hidden layers. This design enables the model to preserve modality-specific distributional characteristics while learning complementary and potentially nonlinear relationships among omics layers in a joint latent space [30,34]. For example, Flexynesis processes each omics matrix through a dedicated encoding network before combining the resulting representations for downstream prediction, thereby reducing the risk that a single high-dimensional modality dominates the integrated model [35]. More complex architectures further incorporate hierarchical fusion. Triad-LMF first models pairwise relationships among modalities through a local pairwise fusion module and subsequently integrates the resulting representations through a global triadic fusion module, enabling the learning of hierarchical cross-modal associations [36]. Similarly, SynOmics combines intra-omics and cross-omics graph learning to model both modality-specific and shared signals, demonstrating the potential value of explicitly representing relationships within and between omics layers [37]. Although intermediate fusion often provides a favorable balance between modality-specific information and cross-modal interaction learning, its performance remains dependent on sample size, data quality, modality complementarity, and the design of the latent representation.

Late fusion adopts a more modular strategy in which a separate predictive model is trained for each omics modality, and the resulting predictions or learned representations are subsequently combined through averaging, weighted voting, or a meta-learning framework [20,34]. This design provides considerable modeling flexibility because each data type can be analyzed using an architecture tailored to its specific characteristics. It can also be advantageous when modalities differ substantially in quality or when some modalities are missing. BiBoNet provides an example of this approach by learning modality-specific representations from metabolomic and microbiome data using dedicated multilayer perceptrons and integrating these representations at a later stage for classification, outperforming naive early fusion in benchmark datasets [29]. However, because information from different modalities is integrated only after substantial modality-specific processing, late fusion may have limited capacity to capture low-level or intermediate cross-modal interactions. Its utility is therefore greatest when modality-specific predictive signals are relatively independent or when robustness and modularity are prioritized over detailed modeling of cross-omics synergy [30].

The fusion strategy defines when and how information is combined, whereas the underlying neural architecture determines which biological patterns can be represented and learned. Different architectures therefore provide distinct inductive biases and are suited to different data structures and biological questions. Autoencoders and variational autoencoders are widely used for dimensionality reduction and representation learning because they can compress high-dimensional and sparse omics data into lower-dimensional latent spaces while retaining informative variation [38,39]. The Deep Latent Space Fusion model employs a cycle deep autoencoder with a shared self-expressive layer to integrate heterogeneous omics data and capture global sample-level structure [40]. Similarly, Deep Subspace Mutual Learning (DSML) uses modality-specific autoencoders to learn latent subspace representations before integrating them for downstream classification [41]. These studies demonstrate that autoencoder-derived latent representations can provide compact and denoised features for phenotype prediction and other downstream tasks. However, latent spaces learned solely from reconstruction objectives may not explicitly encode known molecular interactions, phylogenetic relationships, or ecological dependencies, limiting their ability to represent the relational organization of plant-microbiome systems.

Graph neural networks (GNNs) provide a complementary framework for modeling the relational structure of biological systems. Host-microbiome interactions can be represented through multiple graph types, including host-microbe association networks, microbial co-occurrence networks, phylogenetic relationships, metabolic pathways, and molecular regulatory networks [42,43]. GNNs jointly learn from node attributes and graph topology, enabling the model to incorporate biological context that is not represented in tabular feature matrices. For example, a multi-omics integration model based on graph convolutional network (GCN) combines autoencoder-based dimensionality reduction with graph convolutional learning on sample-similarity networks to improve molecular subtype classification [44]. More advanced frameworks, including SynOmics, model intra- and inter-omics relationships using graph-based architectures, whereas MOGONET applies modality-specific graph convolutional networks to learn complementary multi-omics representations [33,37]. These approaches demonstrate that modeling biological relationships can provide information beyond feature values alone. Nevertheless, GNN performance depends strongly on graph construction, and incomplete, noisy, or context-inappropriate networks may introduce bias or obscure biologically relevant interactions [45,46]. In addition, conventional message-passing architectures can have difficulty capturing long-range dependencies across large and heterogeneous biological graphs.

Transformer architectures have attracted increasing attention because self-attention enables the modeling of long-range dependencies and global contextual relationships. In multi-omics applications, this capability may facilitate the identification of nonlocal associations, such as relationships between distant genomic regions, regulatory genes, microbial taxa, and downstream metabolites [47,48]. Multi-head attention allows the model to dynamically weight information from different features or modalities according to its relevance to a given prediction [47]. For example, ADINet employs attention-based mechanisms to adaptively integrate multi-omics information and estimate the relative contribution of different modalities and features [49,50]. More broadly, large biological foundation models pre-trained on genomic [51], transcriptomic [52], or protein sequences [53] offer opportunities to transfer representations learned from extensive unlabeled datasets to plant and microbial prediction tasks. Such approaches may be particularly valuable for plant-microbiome studies, where labeled multi-omics cohorts are often limited. However, the application of Transformers and foundation models to integrated plant-microbiome datasets remains at an early stage, and their computational requirements, data demands, and biological interpretability require careful evaluation. Thus, although deep learning can enhance the integration of heterogeneous omics data and modeling of complex cross-modal relationships, improved predictive performance alone does not establish biological relevance or mechanism [5,21]. These limitations motivate the development of interpretable and biologically grounded AI frameworks, discussed in the following sections. Where multiple studies were available, representative examples were selected to illustrate both methodological development and biological application. Particular emphasis was placed on distinguishing architectures validated in plant-microbiome systems from methods established primarily in other biological domains.

The use of AI in plant-microbiome research is also constrained by computational and data-governance considerations. Although conventional machine-learning methods are generally accessible with modest computational resources, deep-learning models, graph neural networks, Transformers, and foundation models may require substantial memory, graphics processing unit capacity, and training time, particularly when multiple omics layers are integrated [54]. Lightweight models, transfer learning, and shared or cloud-based computing may improve accessibility, while proprietary breeding materials, farm-level measurements, and commercially generated multi-omics datasets may impose additional restrictions on data privacy, access, and reuse [55].

### 2.3. Current Limitations of Predictive Models

Microbiome abundance data pose a distinctive challenge for AI and XAI because sequencing-based measurements are inherently compositional: relative abundances are constrained by a fixed total and therefore cannot be interpreted independently [56]. This structure can affect correlation patterns, feature selection, predictive modelling, and the biological interpretation of feature attribution [28]. Recent benchmarking studies further indicate that data transformation and abundance representation can substantially influence predictive performance and SHAP-derived microbial biomarkers [57]. Therefore, XAI-derived microbial features should be interpreted in the context of the underlying preprocessing strategy. Appropriate compositional transformations, treatment of sparse and zero-inflated features, prevalence filtering, and, where available, absolute-abundance measurements can improve robustness. Sensitivity analyses across reasonable preprocessing pipelines are also valuable for identifying features whose attribution remains stable across analytical choices. Thus, preprocessing should be considered an integral component of XAI interpretation rather than merely a technical step preceding model development.

Despite the substantial predictive power of machine-learning and deep-learning models for plant-microbiome multi-omics integration, several limitations constrain their capacity to generate biologically meaningful and mechanistically informative knowledge. First, the biological interpretability of many high-performing models remains limited. Although these models can learn complex nonlinear mappings from highly heterogeneous data and achieve strong predictive performance across diverse biological tasks [29], the internal representations underlying their predictions are often difficult to translate into biologically meaningful processes. Consequently, accurate prediction does not necessarily reveal the cross-kingdom regulatory, metabolic, or signaling mechanisms that connect plant hosts, microbial communities, and environmental conditions [58]. Deep architectures have demonstrated strong performance in applications such as antimicrobial-resistance prediction and multi-omics integration [22,59], but increasing model complexity can further obscure how specific biological features and their interactions contribute to model outputs.

Moreover, the biological interpretation of feature importance remains challenging. Even when predictive models identify highly influential microorganisms, genes, or metabolites, these features cannot be assumed to represent biological drivers. In complex plant-microbiome datasets, correlated or functionally related variables may contain overlapping information, causing importance to be distributed across multiple features or assigned preferentially to one representative feature. As a result, model-derived importance rankings may not reliably distinguish putative causal regulators from downstream responses, ecological proxies, or correlated biomarkers. Post hoc explanation methods such as SHAP and LIME can improve the transparency of individual predictions and global model behavior, but their interpretations remain conditional on the trained model, the underlying data distribution, and the assumptions used to generate feature attributions. Feature collinearity, model instability, and the choice of reference or perturbation distributions can therefore affect explanation consistency and may complicate biological interpretation [22,58].

A deeper limitation is the distinction between predictive association and biological mechanism. Most supervised learning models are optimized to identify statistical relationships that improve prediction rather than to infer causal relationships among biological variables. A highly accurate classifier may predict disease resistance or identify reproducible host-microbiome associations without explaining why a particular microbial community suppresses pathogens or promotes plant growth. Nor can predictive importance alone determine whether a feature is an upstream regulator, a downstream consequence, a context-dependent mediator, or a non-causal correlate. Therefore, improvements in predictive accuracy, although valuable, are insufficient to fulfill the broader objective of biological research: understanding how biological systems function and respond to perturbation. Addressing this gap requires XAI frameworks that can make model behavior more transparent, support biologically informed interpretation, and prioritize experimentally testable hypotheses. Importantly, XAI should be viewed not as a direct substitute for mechanistic or causal inference, but as an intermediate step linking predictive models to biological interpretation and experimental validation.

## 3. Explainable AI: From Feature Importance to Biological Insight

### 3.1. Post Hoc Explanation Methods

Post hoc explanation methods interpret the behaviour of trained ML models without modifying their underlying architectures. By quantifying the contribution of individual input features to model predictions, these approaches transform complex predictive models into interpretable biological hypotheses. Depending on the scope of interpretation, post hoc methods provide either global explanations, which provides a comprehensive view of a model’s overall behavior, or local explanations, which offers insights into how individual inputs affect the model’s outputs [17]. In biological research, these approaches enable the conversion of model outputs into candidate genes, microbial taxa, metabolites, and regulatory elements, representing the first step toward moving AI from prediction to mechanism discovery.

Among existing post hoc methods, SHAP, LIME (Local Interpretable Model-agnostic Explanations), and IG (Integrated Gradients) are the most widely adopted approaches because they are model-agnostic or can be readily applied to deep learning models while providing biologically meaningful feature attribution (Figure 1).

#### 3.1.1. SHAP

SHAP is one of the most widely used feature attribution methods in multi-omics research. SHAP derives from cooperative game theory, treating each feature as a “player” in a coalition and fairly distributing the prediction contribution via Shapley values [60]. For feature i, the SHAP value is computed as:

$$\phi_{i}=\sum_{S\subseteq D\setminus \left\{i\right\}}\frac{\left|S\right|!\left(\left|D\right|-\left|S\right|-1\right)!}{\left|D\right|!}\left[f_{S\cup \left\{i\right\}}\left(X_{S\cup \left\{i\right\}}\right)-f_{S}\left(X_{S}\right)\right]$$

where D denotes all features, S is a subset excluding i, and the term in brackets represents the marginal contribution of adding feature i.

SHAP can provide both global explanations, analyzing the key driving factors across the entire dataset, and local explanations, revealing the rationale behind individual sample predictions, making it particularly well-suited for highly heterogeneous biological data [61]. In multi-omics studies, SHAP has been increasingly adopted to prioritize biologically relevant features across different omics layers. Depending on the analytical objective, these features may correspond to candidate genes in transcriptomics, regulatory variants in genomics [62], proteins in proteomics, metabolites in metabolomics, or microbial taxa in microbiome studies [63], thereby facilitating downstream biomarker discovery and mechanistic interpretation. For example, it has been applied to identify key rhizosphere microbial taxa associated with Fusarium wilt resistance, ranking bacterial biomarkers that contributed to plant disease prediction models and providing insights into plant-microbiome interactions [64].

#### 3.1.2. LIME

Unlike SHAP, which provides both global and local explanations, LIME focuses primarily on explaining individual predictions by constructing a simple interpretable surrogate model around a specific sample [65]. Specifically, LIME perturbs the input features around an individual sample and fits a simple linear model to approximate the behavior of the original predictor within that local region.

Local interpretability is particularly valuable in plant-microbiome studies as biological systems often exhibit considerable heterogeneity among genotypes, developmental stages, soil types, and environmental conditions. LIME enables researchers to investigate sample-specific drivers underlying unusual phenotypes or prediction outcomes, thus providing insight into context-dependent biological mechanisms. However, LIME relies on random perturbation and local sampling strategies, and its explanations may vary over repeated analyses, especially when applied to high-dimensional omics datasets characterized by extensive feature correlations [65]. Therefore, LIME is generally considered complementary to SHAP rather than a direct alternative.

#### 3.1.3. IG

IG is a gradient-based attribution method that quantifies feature importance by accumulating gradients along the path from a baseline input to the observed sample, thereby alleviating the gradient saturation problem commonly encountered in conventional saliency maps [66]. Compared with simple gradient-based visualization approaches, IG provides more stable and biologically meaningful feature attributions, making it well suited for interpreting deep learning models in multi-omics research (Figure 1). IG and related gradient-based attribution methods have been widely applied to identify regulatory DNA elements, prioritize functional genomic regions, and interpret transcriptomic and protein sequence models [66,67]. Although their application in plant-microbiome studies is still limited, these approaches show considerable potential for explaining deep neural networks trained on metagenomic, metatranscriptomic, metabolomic, and host transcriptomic data, facilitating the prioritization of key microbial taxa, functional genes, metabolites, and host molecular signatures associated with complex plant traits. Related methods, including DeepLIFT, GradientSHAP, and DeepSHAP, further extend gradient-based interpretation to increasingly complex neural network architectures.

### 3.2. Intrinsically Interpretable Architectures

Unlike post hoc explanation methods that offer explanations after model training, intrinsically interpretable architectures incorporate interpretability directly into the model design by incorporating biological knowledge or transparent computational structures. Such models generate explanations simultaneously with predictions, enabling the direct mapping of model outputs onto biomedical meaningful entities, such as genes, regulatory elements, pathways, microbial taxa, and interaction networks. This design is particularly advantageous for plant-microbiome multi-omics studies with complex cross-kingdom interactions that require accurate prediction and mechanistic interpretation.

Attention mechanisms are one of the most frequently used intrinsically interpretable techniques. Attention layers allow the models to identify the most influential genes, genomic regions, proteins, or microbial taxa for a given prediction by assigning adaptive weights to the input features. Transformer based architectures have shown remarkable ability in modelling long range chromatin interactions in regulatory genomics. For example, Although Enformer, BPNet, and DeepSEA were developed primarily for genomic applications outside the plant-microbiome field, they illustrate the capacity of attention- and sequence-based architectures to capture biologically meaningful regulatory patterns [62,68,69]. Similarly, DCell and PASNet were developed mainly in biomedical and cancer-related contexts, where they demonstrate the potential of biologically informed neural networks to incorporate pathway structure, gene-phenotype relationships, and prior biological knowledge into predictive modelling [70,71]. However, the applications of attention-based sequence models and biologically constrained neural networks to integrated plant-microbiome multi-omics remains comparatively limited. The examples discussed here should therefore be interpreted primarily as methodological evidence from broader biological domains, rather than as evidence that these architectures have already been comprehensively validated in plant-microbiome systems. Their use in plant-microbiome systems will require adaptation to plant-specific regulatory networks, microbial community structure, host-microbe interactions, and heterogeneous multi-omics data.

Another important class of intrinsically interpretable models consists of biologically informed sparse neural networks. Instead of learning unrestricted feature interactions, these models constrain network connectivity according to prior biological knowledge, such as Gene Ontology (GO) hierarchies, signaling pathways, or gene regulatory networks. DCell organized hidden neurons according to hierarchical cellular functions, allowing each computational module to correspond to a biological process [70]. Similarly, PASNet restricted neural connections using curated pathway information, enabling pathway-level interpretation while maintaining competitive predictive accuracy [71]. Such biologically constrained architectures improve model transparency by transforming latent representations into interpretable biological modules, making them particularly suitable for multi-omics integration where prior biological knowledge is abundant.

Beyond individual molecular features, graph-based interpretable architectures provide an effective framework for modeling the highly interconnected nature of plant holobiont systems. Plant-associated microbiomes consist of complex ecological networks in which microbial taxa interact with one another and with host plants through multiple molecular and environmental processes. GNNs naturally capture these dependencies by representing biological entities as nodes and their interactions as edges, while graph attention mechanisms further identify influential nodes and interaction pathways responsible for model predictions. Compared with conventional ML models that treat microbial taxa independently, graph-based approaches preserve the ecological structure of microbial communities and therefore offer greater biological interpretability.

Graph-based and attention-based architectures are increasingly relevant to plant-microbiome research, although direct applications remain less extensive than those reported in other biological domains. Ecological network analyses have successfully reconstructed microbial interaction networks and identified keystone taxa that regulate community stability and ecosystem functioning [72]. Studies of root-associated fungal communities further demonstrated that plant-fungus associations exhibit highly modular network organization, indicating that ecological interactions rather than individual species abundances govern microbiome assembly [73]. More recently, network analyses of the *Arabidopsis thaliana* root microbiome showed that host genetic variation reshapes microbial interaction networks, highlighting the importance of considering network topology when investigating host-microbe relationships [74]. These findings suggest that intrinsically interpretable architectures are particularly well suited for plant-microbiome multi-omics because they can reveal not only predictive biomarkers but also the ecological and molecular interactions underlying host-microbe communication. As graph learning, biologically informed neural networks, and attention mechanisms continue to evolve, they are expected to play an increasingly important role in transforming predictive models into tools for mechanistic biological discovery.

### 3.3. Mapping AI Explanations to Biological Entities

Although XAI methods can identify the features that drive model predictions, feature importance alone does not provide biological understanding. The ultimate objective of XAI in multi-omics research is therefore to translate computational explanations into biologically meaningful entities, such as genes, proteins, metabolites, microbial taxa, regulatory elements, and biological pathways [75].

This translation process commonly integrates feature attribution with prior biological knowledge. Candidate genes identified by SHAP, Integrated Gradients or DeepLIFT are frequently annotated using GO, Kyoto Encyclopedia of Genes and Genomes (KEGG), Reactome, MetaCyc and other biological databases to reveal their molecular functions and associated biological processes [76,77]. Pathway enrichment analyses further determine whether highly ranked features converge on coordinated biological functions, such as phytohormone signalling, nutrient metabolism, immune regulation or secondary metabolite biosynthesis. Compared with interpreting individual biomarkers, pathway-level analyses provide a more robust understanding of biological regulation because complex phenotypes generally arise from coordinated activities of multiple genes and molecular pathways rather than isolated components [78]. More importantly, these computationally prioritized candidates frequently become targets for downstream transcriptomic analyses, functional genomics and gene-editing experiments, allowing AI-derived explanations to be validated experimentally.

Beyond pathway annotation, network-based interpretation has become an increasingly important strategy for translating AI explanations into mechanistic insights. Cellular functions emerge through complex interaction networks involving gene regulatory interactions, protein–protein interaction, metabolic reactions, signaling pathways, and microbial ecological relationships [79]. Integrating feature importance with these biological networks enables the identification of regulatory hubs, functional modules, and key signaling cascades that may not be evident from feature ranking alone. For example, genes with only moderate attribution scores may represent essential regulators if they occupy central positions within highly connected network modules, whereas highly ranked but isolated features may have limited biological significance. Network topology therefore provides complementary information that enhances the interpretation of AI-derived explanations [80].

The interpretation process becomes substantially more challenging in multi-omics studies because biologically meaningful information is distributed across multiple molecular layers. Gene expression influences protein abundance and metabolite accumulation, while microbial communities interact dynamically with host metabolism and immune signaling. Therefore, biologically relevant explanations require integrating attribution results across genomics, transcriptomics, proteomics, metabolomics, and microbiome datasets [81]. Recent advances increasingly combine explainable AI with graph neural networks, knowledge graphs, and heterogeneous biological networks to model these cross-omics interactions explicitly [82]. Rather than identifying isolated biomarkers, these approaches reveal coordinated regulatory modules spanning multiple omics layers, thereby providing a systems-level understanding of biological processes.

This hierarchical interpretation strategy is particularly valuable for plant-microbiome systems. Plant growth and adaptation are governed by continuous interactions among host genetic regulation, microbial community assembly, metabolite exchange, and environmental conditions [83]. Explainable AI can simultaneously identify host genes involved in immune responses or nutrient uptake, microbial taxa associated with plant health, and metabolites functioning as signaling molecules during host-microbe communication. Mapping these biological entities onto host-microbiome interaction networks allows researchers to distinguish direct regulatory mechanisms from indirect ecological associations. Moreover, incorporating prior biological knowledge into AI interpretation improves confidence in identified interactions and facilitates the generation of biologically plausible hypotheses for subsequent experimental validation.

Overall, the value of XAI extends beyond improving model transparency. By systematically mapping computational explanations onto genes, regulatory elements, metabolites, microbial functions, and cross-kingdom interaction networks, XAI is evolving from a model interpretation tool into a hypothesis-generation framework that accelerates biological discovery in plant-microbiome multi-omics research.

## 4. From Biological Insight to Mechanistic Discovery

### 4.1. Biological Insights Enabled by Explainable AI

XAI does not directly uncover biological mechanisms; rather, it provides biologically interpretable evidence by revealing informative features, interactions, and regulatory patterns underlying model predictions. These biological insights span multiple levels of biological organization, from individual molecules and pathways to microbial communities and cross-kingdom interaction networks, thereby providing a framework for transforming predictive models into experimentally testable biological hypotheses.

At the molecular scale, XAI identifies candidate biomarkers by quantifying the contribution of individual features to model predictions. The LP-Micro framework exemplifies this approach by employing polynomial group lasso to characterize longitudinal microbial abundance trajectories and permutation-based statistical tests to estimate the significance of taxon- and time-specific effects. This strategy effectively accommodates the sparsity and zero inflation typical of microbiome datasets while prioritizing microbial taxa that contribute most strongly to disease prediction at different developmental stages [84]. Beyond individual biomarkers, XAI can also identify key regulatory components within molecular networks. By integrating transcriptomic, proteomic, and metabolomic datasets, recent studies have reconstructed dynamic regulatory networks underlying plant-microbe-insect interactions and identified master regulators such as CIPK23, a central hub in drought signal transduction, together with immune signaling modules involving calcium signaling, protein kinase cascades, and hormone crosstalk [85].

Beyond individual taxa and pathways, XAI increasingly enables the interpretation of cross-kingdom interaction modules that connect plant genomes, microbial communities, metabolites, and environmental responses. Recent advances in structural deep learning, including AlphaFold-Multimer and protein language models, are creating new opportunities for the identification of structurally conserved but sequence-unrelated pathogen effectors. By predicting host–pathogen protein–protein interaction interfaces at residue-level resolution, these approaches can provide structurally interpretable evidence for how microbial effectors manipulate host immune systems [86]. Complementing structural prediction, SHAP interaction analysis has been used to construct cross-omics association networks that reveal coordinated interactions among plant metabolites such as daidzin, signaling molecules including GABA, and microbial taxa such as *Paenibacillus* during drought stress [25]. Likewise, attention-based models have identified regulatory hotspots within ABA (abscisic acid)-associated metabolic pathways while simultaneously revealing interaction modules linking rhizosphere microbiomes, ABA signaling, and systemic drought responses [87]. Together, these studies demonstrate that XAI can integrate information across multiple biological layers to identify candidate interaction modules that would be difficult to detect using conventional statistical analyses alone.

Collectively, these studies demonstrate that XAI can transform opaque model outputs into biologically interpretable hypotheses spanning molecular, pathway, community, and cross-kingdom levels of biological organization. Importantly, however, the biological entities and interactions identified by XAI remain computational inferences rather than evidence of biological causality. Whether these predictions represent genuine regulatory mechanisms ultimately depends on rigorous experimental validation. Consequently, the greatest value of XAI lies not in replacing experimental biology, but in prioritizing biologically plausible hypotheses that can guide targeted validation and accelerate mechanistic discovery.

### 4.2. Experimental Validation of AI-Derived Biological Hypotheses

To facilitate comparison across studies, Table 1 summarizes representative applications of AI and XAI in plant-microbiome research, including the biological system, omics layers, predictive models, explanation approaches, prioritized biological candidates, and experimental validation. Importantly, the table also distinguishes computational prioritization from experimentally supported biological evidence.

Despite the growing ability of explainable AI to identify candidate biological entities across multiple organizational scales, the ultimate value of these predictions depends on whether they can be experimentally validated and translated into mechanistic understanding. Recent studies increasingly demonstrate that AI-generated hypotheses can guide experimental design, accelerate biological discovery, and reveal causal relationships within plant-microbiome systems.

At the microbial community level, AI-derived predictions have successfully identified keystone taxa that regulate ecosystem functioning. Using neural ordinary differential equations, the DLKTI framework inferred microbial community dynamics and quantified the structural importance of individual taxa through in silico perturbation. Applied to submerged macrophyte rhizosphere microbiomes, DLKTI identified *Paludibaculum* and *Desulfobulbus* as keystone genera. Subsequent partial least squares path modeling demonstrated that the α-diversity of these predicted keystone taxa was significantly associated with microbial biomass phosphorus, whereas their β-diversity influenced plant functional traits that ultimately determined phosphorus homeostasis and phytoplankton growth inhibition. Together, these analyses established a mechanistic cascade linking AI-predicted keystone microbes to ecosystem-level phosphorus cycling [88].

Complementary evidence has emerged from gene discovery and functional validation. An evolutionarily informed XGBoost framework used nitrogen-responsive genes conserved between *Arabidopsis* and maize as biologically informed features, substantially improving nitrogen-use efficiency prediction compared with randomly selected genes (maize: r = 0.79 versus 0.62). Feature attribution prioritized NF-YA6, NLP5, and WRKY38 as candidate regulators of nitrogen-use efficiency. Importantly, these computational predictions were validated using *Arabidopsis* T-DNA insertion mutants, maize UniformMu mutants, and field experiments, in which the maize nfya3-1::Mu mutant exhibited significantly improved nitrogen-use efficiency [89]. Likewise, rhizosMASH combined profile hidden Markov models and Random Forest models with bacterial genomic profiles to predict rhizosphere competence from catabolic gene clusters, accurately identifying genomic determinants of root colonization in both *Arabidopsis* and potato [90]. These studies illustrate how AI can prioritize candidate genes and microbial functions for targeted experimental verification.

AI-guided predictions have also advanced understanding of disease-suppressive microbiomes. By integrating microbiome data from 25 independent studies, a Random Forest model identified 54 biomarker genera, including *Bacillus*, *Nocardioides*, and *Kribbella*, that were consistently enriched in disease-suppressive soils [91]. Successive microbiome passaging experiments in the *Arabidopsis-Pseudomonas* syringae pathosystem confirmed progressive disease suppression accompanied by enrichment of these taxa. Transcriptomic analyses further demonstrated that the enriched microbial consortium enhanced pattern-triggered immunity through activation of reactive oxygen species production and mitogen-activated protein kinase signaling rather than direct pathogen antagonism, thereby revealing the biological mechanism underlying AI-prioritized microbial biomarkers.

Beyond disease resistance, explainable AI has also facilitated the interpretation of beneficial plant-microbe interactions in crop production. A spatiotemporal convolutional neural network coupled with SHAP analysis identified root colonization, specific root length, photosynthetic capacity, and plant nitrogen status as the principal determinants of maize yield under arbuscular mycorrhizal fungi (AMF) and plant growth-promoting rhizobacteria inoculation [92]. Counterfactual analysis further suggested that AMF-mediated improvements in root architecture contributed disproportionately to yield under favorable nitrogen conditions, generating experimentally testable hypotheses regarding context-dependent microbial benefits.

Collectively, these studies illustrate an important transition in plant-microbiome research, from using AI solely for prediction to employing it as a hypothesis-generation engine that guides mechanistic experimentation. Rather than replacing experimental biology, AI is increasingly functioning as a discovery platform that prioritizes candidate genes, microbial taxa, metabolic pathways, and cross-kingdom interactions for targeted validation. The integration of explainable AI with functional genomics, microbial ecology, and controlled perturbation experiments therefore provides a practical route for moving from computational predictions torward mechanistic understanding. Current studies span a broad range of evidence levels, but relatively few have progressed from XAI-based candidate prioritization to controlled perturbation and mechanistic validation (Table 1). Moreover, fully iterative cycles in which experimental findings are incorporated into subsequent model training remain uncommon [93].

**Table 1:** Representative plant-microbiome studies using AI/XAI and their levels of biological and experimental validation.

Study	Plant/Host	Microbiome or Interaction Context	Omics/Data Layers	AI Model	XAI/Interpretability Approach	Biological Candidates or Outputs	Experimental/Biological Validation
Yoshioka et al. [25]	Soybean	Rhizosphere microbiome	Host, microbial, metabolomic, and environmental data	Random Forest; XGBoost	SHAP	Plant metabolites; Microbial taxa	\
Su et al. [64]	Tomato	Rhizosphere microbiome associated with *Fusarium* wilt resistance	Microbiome	Random Forest	SHAP	Key rhizosphere bacterial taxa	Microbial in vitro antagonism; in planta biocontrol assay
Pan et al. [88]	Submerged macrophytes	Rhizosphere microbiome	Microbial community dynamics; Plant functional traits	Deep learning/neural ordinary differential equation framework	In silico taxon-removal/keystoneness analysis	Keystone microbial taxa	Relationships between predicted keystone taxa and plant traits
Li et al. [90]	Arabidopsis, potato	Plant-associated bacteria	Bacterial genomic profiles/catabolic gene clusters	Profile hidden Markov models; Random Forest	Rule-based genome mining tool	Bacterial gene clusters	Comparison against curated datasets
Zhang et al. [91]	Arabidopsis	Disease-suppressive microbiomes	Microbiome data; Plant transcriptomics	Random Forest	Model-based biomarker prioritization	Key biomarker genera	Microbial inoculation assay; Plant transcriptomics; Induced systemic resistance
Jahan & Nassiri-Mahallati [92]	Maize	AMF and plant growth-promoting rhizobacteria	Plant physiology; Microbial inoculation	Convolutional neural network	SHAP; LIME	Key plant traits	Counterfactual analysis

### 4.3. From Association to Causality: The Remaining Challenge

Although XAI has greatly improved the biological interpretability of predictive models, a fundamental challenge remains: interpretable associations do not constitute causal evidence [94]. XAI can identify features, interactions, or pathways that contribute strongly to model predictions, but these contributions reflect their influence on model performance rather than their causal effects on biological phenotypes [95]. Whether quantified by SHAP values, attention weights, or permutation importance, feature attribution measures explain how a model makes predictions, not how biological systems function. Failure to distinguish these concepts risks overinterpreting computational explanations as mechanistic discoveries [96].

Recent evidence highlights the preprocessing-dependent instability for SHAP-based feature attribution in microbiome classification. A systematic benchmarking analysis comparing eight data-transformation methods across 24 metagenomic datasets found that similar predictive performance could be accompanied by substantially different top-ranked features [97]. For RF models, the overlap among the top 25 predictors derived from presence-absence and abundance-based transformations was approximately 50%, indicating that feature importance can depend strongly on analytical representation rather than solely on underlying biology. However, the stability of other explanation methods, including LIME, IG, and attention-based approaches, remains insufficiently benchmarked in plant-microbiome multi-omics. Explanation stability across models, preprocessing strategies, reference choices, and biological contexts therefore remains an important methodological gap.

The challenge is compounded by feature redundancy and dataset dependency. The same benchmarking study showed that models trained with only the 10–25 highest-ranked microbial features often achieved predictive performance comparable to models using the complete feature set, whereas incorporating additional “important” features frequently reduced performance, suggesting that some attributed features primarily reflect redundancy, feature collinearity, or model-specific artifacts rather than independent biological signals [97]. Furthermore, a recent review emphasized that differences in sampling strategies, sequencing depth, taxonomic annotation, and bioinformatic pipelines can substantially alter feature distributions across studies, thereby limiting the reproducibility and transferability of XAI-derived explanations [98]. Consequently, biomarkers prioritized in one dataset may not be reproduced in independent cohorts, even when addressing similar biological questions.

These limitations underscore an important conceptual distinction. XAI provides explanations of predictive models, not direct evidence of biological causation. Consequently, XAI should be regarded as a hypothesis-generation framework that prioritizes candidate genes, microbial taxa, pathways, and interaction modules for subsequent investigation rather than as a standalone tool for mechanistic inference [96]. Establishing causality ultimately requires experimental perturbation, including genome editing, mutant analysis, synthetic microbial communities, isotope tracing, and other controlled validation strategies capable of testing predicted biological functions [99]. Integrating explainable AI with causal inference and experimental biology therefore represents the critical next step toward transforming computational associations into mechanistic understanding [100].

Causal-inference frameworks can extend XAI from association toward directional biological hypotheses. Structural causal models and causal Bayesian networks can represent relationships among host genotype, environmental conditions, microbial communities, metabolites, and host phenotypes [101]. Invariant causal prediction can identify relationships that remain stable across soils, seasons, genotypes, or environments, while counterfactual inference can estimate phenotypic changes following hypothetical microbial or metabolite interventions [102]. These methods remain emerging in plant-microbiome multi-omics, and their reliability depends on causal assumptions, experimental design, environmental variation, and data quality.

### 4.4. A Closed-Loop Framework for AI-Guided Mechanism Discovery

The preceding sections demonstrate that XAI substantially improves the biological interpretability of predictive models by prioritizing candidate genes, metabolites, microbial taxa, and interaction modules across multiple levels of biological organization. The case studies discussed above further illustrate that AI-derived hypotheses can successfully guide experimental validation and facilitate biological discovery.

Nevertheless, a fundamental challenge remains: XAI provides biologically interpretable associations rather than direct evidence of causality. As emphasized in recent perspectives on phytopathogen effector biology, AI primarily accelerates hypothesis generation, whereas mechanistic understanding ultimately depends on experimental validation in biologically relevant systems [86]. Bridging this gap requires a systematic framework that explicitly integrates computational prediction with iterative experimental verification.

We therefore propose a closed-loop framework for AI-guided mechanism discovery in plant-microbiome systems (Figure 2). Within this framework, heterogeneous multi-omics datasets—including metagenomics, transcriptomics, metabolomics, proteomics, and phenomics—are first integrated to train predictive AI models (Figure 2a,b). XAI methods, such as SHAP, attention mechanisms, and LIME, are subsequently applied to identify the genes, metabolites, microbial taxa, and interaction modules that contribute most strongly to model predictions (Figure 2c). Importantly, XAI is not intended to determine biological function directly; rather, it serves as a hypothesis-generation framework that prioritizes biologically plausible candidates for downstream investigation. Candidates that exhibit consistent importance across multiple models, datasets, or environmental conditions can then be prioritized for experimental validation (Figure 2d,e).

This strategy is consistent with recent advances in systems biology, where integrating multi-omics data with predictive modeling has accelerated the identification of regulatory components and improved multiscale phenotype prediction [103]. However, as these authors also emphasize, the biological impact of computational predictions ultimately depends on their successful integration into experimental validation pipelines.

Experimental validation represents the critical transition from statistical association to mechanistic inference [104] (Figure 2e). Candidate regulatory genes can be validated through CRISPR/Cas-mediated genome editing or classical mutant analyses, whereas predicted keystone microbial taxa can be evaluated using synthetic microbial community drop-out and add-back experiments [105,106]. Likewise, metabolite supplementation, isotope tracing, and targeted functional assays can verify the predicted roles of signaling molecules and metabolic pathways. Rather than serving merely as an endpoint, these experiments generate new biological evidence that can be incorporated into subsequent rounds of model development, enabling iterative refinement of both computational predictions and biological hypotheses (Figure 2f). Such feedback transforms the relationship between AI and experimentation from a linear workflow into a dynamic learning cycle [107]. However, the closed-loop framework proposed here should be considered a conceptual research framework rather than a workflow that has already been implemented end-to-end across plan-microbiome systems. Existing studies have primarily demonstrated individual components of the proposed loop, particularly prediction and explanation, candidate prioritization, and subsequent experimental validation. In contrast, iterative incorporation of experimental results into model retraining and subsequent hypothesis generation remains comparatively underdeveloped.

Ultimately, this closed-loop framework redefines the role of AI in plant-microbiome research. Instead of functioning solely as a predictive tool, AI becomes a hypothesis-driven discovery engine that continuously integrates computational inference with experimental evidence (Figure 2). By coupling multi-omics integration, explainable AI, causal validation, and iterative model refinement, this framework provides a practical pathway from predictive association to mechanistic understanding. More broadly, it aligns with the emerging vision of translational systems biology for next-generation crop improvement, in which data generation, computational modeling, experimental validation, and field deployment form an integrated continuum for developing resilient and sustainable agricultural systems [103].

## 5. Future Perspectives and Conclusions

### 5.1. Toward AI-Enabled Mechanistic Biology

Artificial intelligence has already transformed plant-microbiome research by enabling large-scale integration of heterogeneous omics datasets and improving the prediction of complex phenotypes. However, the next stage of AI development will be defined not by incremental improvements in predictive accuracy, but by its capacity to generate biologically meaningful and mechanistically testable hypotheses. Achieving this transition requires AI systems that incorporate biological knowledge, explicitly model causal relationships, and interact iteratively with experimental validation.

One promising direction is knowledge-guided learning, in which prior biological knowledge is embedded directly into model construction [108]. Rather than treating genes, metabolites, and microbial taxa as independent variables, knowledge-guided approaches incorporate gene regulatory networks, metabolic pathways, phylogenetic relationships, and ecological interactions as structural constraints during model training [109,110]. Such biologically informed architectures reduce the search space of possible solutions, improve model robustness under limited sample sizes, and generate predictions that are inherently more consistent with established biological principles [111]. For plant-microbiome systems, integrating prior knowledge from host signaling pathways, microbial metabolism, and host-microbe interaction networks may substantially improve both predictive performance and biological interpretability.

Closely related to this concept is the development of biology-informed neural networks (BINNs) [112]. Unlike conventional deep-learning models that learn latent representations solely from data, BINNs encode known biological hierarchies directly into network architecture. Neurons and network layers can represent genes, pathways, regulatory modules, or microbial guilds, allowing information to propagate along biologically meaningful connections. Such architectures facilitate interpretation because model outputs can be naturally mapped onto known biological processes instead of abstract latent features. As increasingly comprehensive plant regulatory networks and microbiome interaction databases become available, BINNs are expected to bridge the gap between high-dimensional data integration and mechanistic systems biology [113].

Nevertheless, biological interpretation alone remains insufficient for establishing mechanism. A major future direction is therefore the integration of causal machine learning with explainable AI. Current XAI methods primarily identify statistical associations that explain model behavior, whereas causal inference seeks to estimate how perturbations of individual biological components influence downstream phenotypes. Emerging approaches—including structural causal models [114,115], causal Bayesian networks [116], invariant causal prediction [117], and counterfactual inference—offer opportunities to distinguish true regulatory drivers from correlated biomarkers. Combined with perturbation experiments such as CRISPR/Cas-mediated genome editing, synthetic microbial communities, and metabolite supplementation, causal machine learning may enable AI systems to move beyond feature prioritization toward identifying causal regulatory pathways within plant holobionts.

Another rapidly emerging direction is the incorporation of knowledge graphs into AI frameworks [118,119]. plant-microbiome systems involve heterogeneous entities—including genes, proteins, metabolites, microbial taxa, phenotypes, environmental variables, and agronomic practices—that interact through complex and dynamic relationships. Knowledge graphs provide a unified representation of these diverse biological entities and their interactions, allowing AI models to perform reasoning across multiple biological scales. By integrating literature-derived knowledge with experimental multi-omics datasets, graph-based AI systems may identify previously unrecognized links between host signaling, microbial functions, and environmental responses. More importantly, knowledge graphs provide an ideal foundation for combining data-driven learning with biological reasoning, thereby enhancing both interpretability and hypothesis generation [118].

Ultimately, future AI systems should evolve from predictive algorithms into iterative discovery platforms. Rather than operating independently of biological experimentation, AI should continuously interact with experimental data in a closed feedback loop in which computational predictions guide experiments, experimental observations refine models, and updated models generate increasingly accurate biological hypotheses. Such a framework has the potential to transform AI from a statistical prediction tool into a central component of mechanistic biological discovery.

### 5.2. Toward Interpretable Plant Holobiont Models

Although most current studies focus on individual omics layers or specific host-microbe interactions, plant performance ultimately emerges from the coordinated behavior of the entire plant holobiont. Developing interpretable plant holobiont models therefore represents an essential next step toward predictive and mechanistic plant biology.

Future models should integrate multiple sources of biological information, including host genomic, transcriptomic, proteomic, metabolomic, and phenomic data, together with microbial taxonomic composition, microbial functional potential, community dynamics, and environmental variables such as soil physicochemical properties, climate, and agricultural management practices [120]. Furthermore, AI/XAI applications in plant-microbiome research must expand beyond current models to a wider host range. This limited host coverage hampers generalization, given that host traits shape both microbiome assembly and model-derived interpretations. Rather than treating these datasets as isolated inputs, next-generation AI models should explicitly represent the hierarchical interactions among host genotype, microbial ecology, and environmental context across spatial and temporal scales.

More broadly, the future of plant-microbiome research lies not in constructing increasingly complex AI models, but in building biologically interpretable digital representations of the plant holobiont that integrate prediction, explanation, and mechanistic understanding within a unified computational framework.

### 5.3. Conclusions and Outlook

Artificial intelligence is fundamentally reshaping plant-microbiome research by enabling the integration of heterogeneous multi-omics datasets, improving phenotype prediction, and uncovering biologically meaningful patterns that were previously inaccessible through conventional analytical approaches. As reviewed here, advances in machine learning, deep learning, explainable AI, and multimodal integration have substantially expanded our ability to identify candidate genes, metabolites, microbial taxa, and cross-kingdom interaction modules across multiple levels of biological organization.

However, prediction alone does not constitute biological understanding. The central challenge for the next generation of AI-driven plant biology is not simply to develop more accurate predictive models, but to establish a rigorous framework that links explainable AI, causal inference, and experimental validation. Only through this integration can computational associations be transformed into mechanistic knowledge that explains how plant holobionts function, respond to environmental perturbations, and ultimately determine plant health and productivity.

Looking forward, the convergence of multi-omics technologies, knowledge-guided AI, causal machine learning, and iterative experimental validation may accelerate the transition from data-driven prediction to experimentally supported mechanistic understanding. In this emerging paradigm, AI will no longer serve merely as a tool for analyzing biological data, but as a collaborative engine for generating, testing, and refining biological hypotheses. Such a transition will provide the conceptual and technological foundation for precision microbiome engineering, climate-resilient crop improvement, and the development of sustainable agricultural systems in the era of intelligent biology.

## Figures and Tables

**Figure 1:**
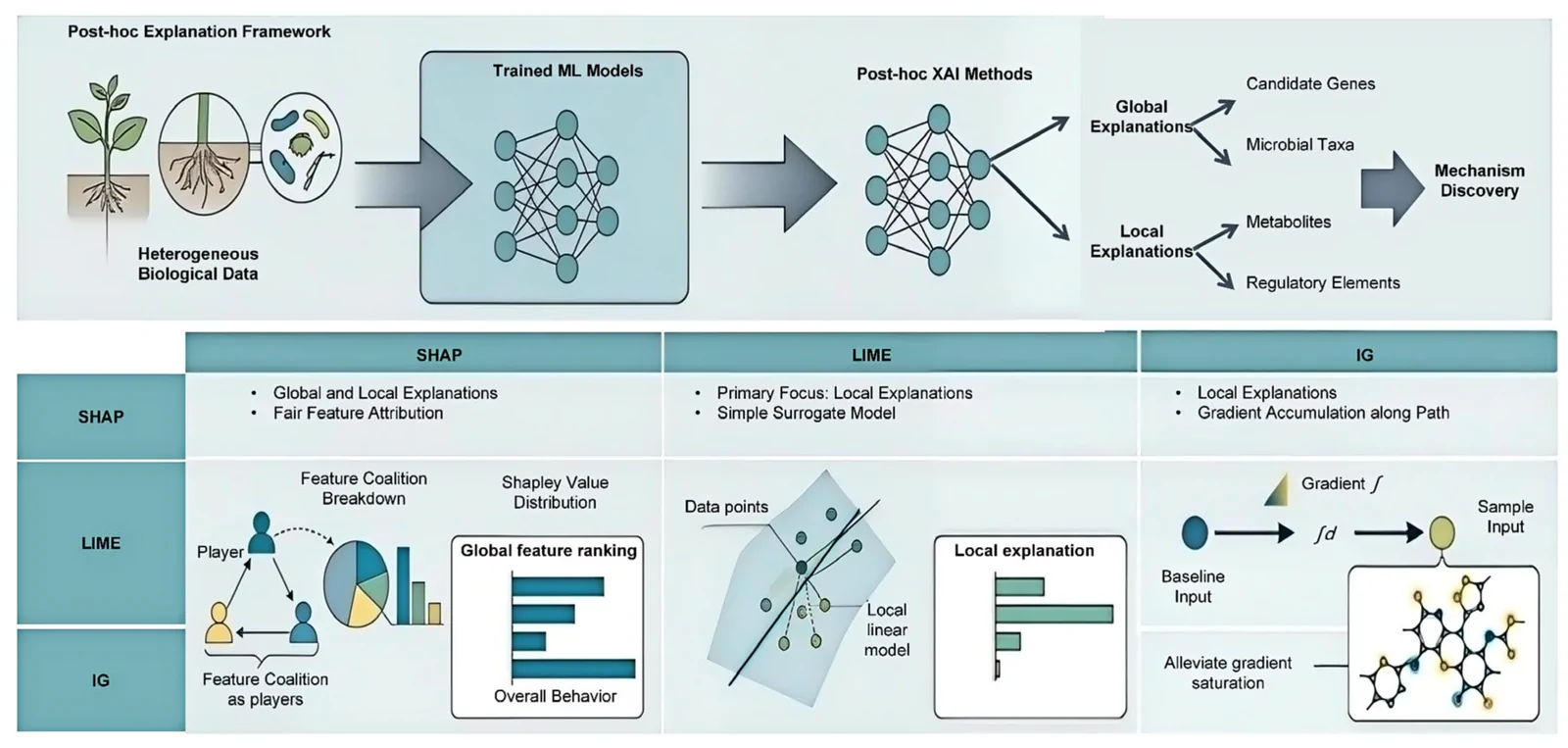
Overview and comparison of post hoc XAI methods in plant-microbiome research. Trained machine learning (ML) models process multi-omics data to generate predictions. Post hoc explainable AI (XAI) methods interrogate these models without altering their architectures, yielding global and local feature attributions that prioritize candidate genes, microbes, and metabolites for biological hypothesis generation. Among key methods, SHAP uses game-theoretic Shapley values for consistent global rankings and local explanations; LIME fits simple local surrogate models by perturbing input data; and Integrated Gradients (IG) integrates gradients from a baseline to overcome gradient saturation in deep neural networks.

**Figure 2:**
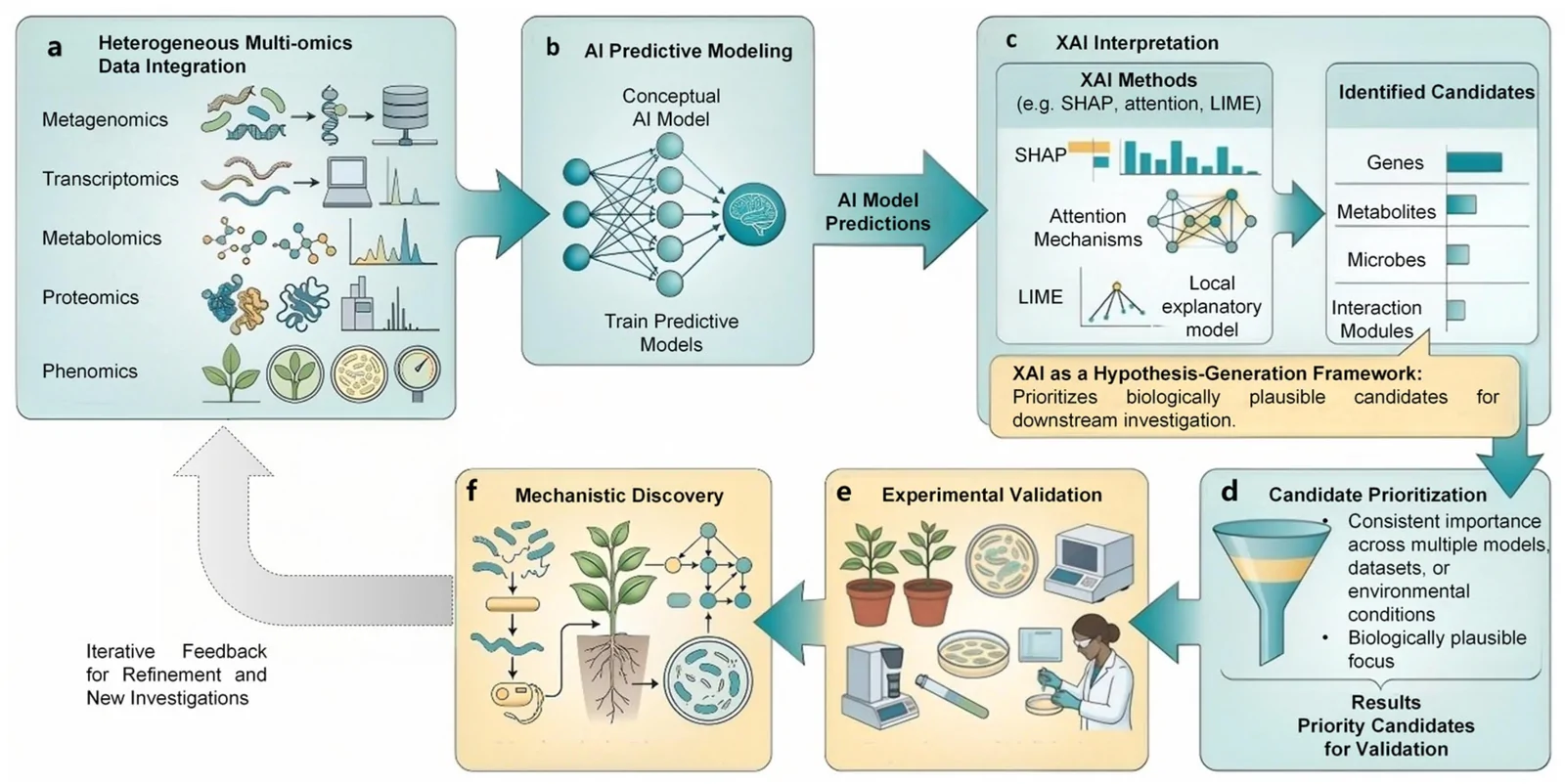
A closed-loop framework for AI-guided mechanism discovery in plant-microbiome systems. (**a**,**b**) Multi-omics datasets (metagenomics, transcriptomics, metabolomics, proteomics, and phenomics) are integrated to train predictive AI models. (**c**) Explainable AI (XAI) techniques (e.g., SHAP, attention mechanisms, LIME) interpret model predictions, functioning as a hypothesis-generation engine to prioritize biologically plausible genes, metabolites, microbes, and interaction modules. (**d**,**e**) Top candidates with consistent importance across datasets or conditions are prioritized for experimental validation (e.g., wet-lab assays, genetic perturbations). (**f**) Validated candidates uncover mechanistic insights into plant-microbiome networks. Solid arrows denote established workflow components; the grey dashed arrow highlights the proposed iterative feedback loop for model refinement, which remains underdeveloped in current research.

## Data Availability

The original contributions presented in this study are included in the article. Further inquiries can be directed to the corresponding author.

## Abbreviations

The following abbreviations are used in this manuscript:

ABA	Abscisic Acid
AI	Artificial Intelligence
AMF	Arbuscular Mycorrhizal Fungi
BINN	Biology-Informed Neural Network
DL	Deep Learning
DLKTI	Deep Learning-based Keystoneness Taxa Identification
DSML	Deep Subspace Mutual Learning
GABA	Gamma-Aminobutyric Acid
GCN	Graph Convolutional Network
GNN	Graph Neural Network
GO	Gene Ontology
IG	Integrated Gradients
KEGG	Kyoto Encyclopedia of Genes and Genomes
LASSO	Least Absolute Shrinkage and Selection Operator
LIME	Local Interpretable Model-Agnostic Explanations
ML	Machine Learning
RF	Random Forest
SHAP	Shapley Additive Explanations
SVM	Support Vector Machine
XAI	Explainable Artificial Intelligence
XGBoost	eXtreme Gradient Boosting
